# Factors Affecting Sleep Quality and Prenatal Distress Among Rural and Urban Women During Early Pregnancy

**DOI:** 10.7759/cureus.69566

**Published:** 2024-09-16

**Authors:** Mugdha Deshpande, Neha Kajale, Nikhil Shah, Anagha Pai Raiturker, Sanjay Gupte, Leena Patankar, Jasmin Bhawra, Shilpa Yadav, Tarun Reddy Katapally, Anuradha Khadilkar

**Affiliations:** 1 Department of Growth and Pediatric Endocrinology, Hirabai Cowasji Jehangir Medical Research Institute, Pune, IND; 2 Department of Health Sciences, Savitribai Phule Pune University, Pune, IND; 3 Department of Pediatric Endocrinology, MRR Children’s Hospital, Mumbai, IND; 4 Department of Obstetrics and Gynecology, Pai Raiturker Clinic, Pune, IND; 5 Department of Obstetrics and Gynecology, Gupte Hospital, Pune, IND; 6 Department of Obstetrics and Gynecology, Patankar Hospital, Pune, IND; 7 School of Occupational and Public Health, Toronto Metropolitan University, Toronto, CAN; 8 Department of Pediatrics, Jehangir Hospital, Pune, IND; 9 Digital Epidemiology and Population Health Laboratory (DEPtH Lab) School of Health Studies, Faculty of Health Sciences, Western University, London, Ontario, CAN; 10 Department of Epidemiology and Biostatistics, Schulich School of Medicine and Dentistry, Western University, London, Ontario, CAN

**Keywords:** bmi, early pregnancy, maternal health, prenatal distress, sleep quality

## Abstract

Background

Early pregnancy is characterized by the initiation of physiological and psychological changes, which places pregnant women at risk of psychological distress and poor sleep, which is known to cause adverse maternal and neonatal outcomes. This study aimed to assess the prevalence of prenatal distress and sleep quality during early pregnancy and identify factors associated with prenatal distress among pregnant women from urban and rural settings.

Methods

The study was conducted with 325 pregnant women (175 rural, 150 urban) as a baseline assessment of the MAI (Mother and Infant) cohort, a longitudinal observational study in Pune, India. Data on sociodemography, anthropometry, clinical history, prenatal distress, and sleep quality were collected between August 2020 and March 2023. Mann Whitney U test and regression were used to assess correlates of sleep quality and prenatal distress.

Results

Over one-third (37.5%) (n=122) of women experienced prenatal distress. Women from rural areas reported a higher prevalence (40%) (n=70) of distress, and poorer sleep quality than urban women (51.4% (n=90) vs 38.7% (n=58)). High prenatal distress was moderately associated with poor sleep quality (ρ = 0.308, *p* = 0.001). After controlling for sociodemographic and clinical factors, high prenatal distress (B=2.63, 95% CI: 1.47-4.69) predicted poor sleep quality. Rural residence (OR: 6.37, 2.46-16.51), underweight BMI status (OR: 2.21, 0.97-5.05), presence of episodes of vomiting (OR: 1.70, 0.93-3.13), and poor sleep quality (OR: 0.74, 0.40-1.38) significantly (*p*<0.05) contributed to prenatal distress.

Conclusion

Prenatal distress and poor sleep quality are significant concerns for pregnant mothers globally and require early screening and management strategies to avoid adverse maternal and fetal outcomes.

## Introduction

Pregnancy is characterized by physiological and psychological alterations that may result in pregnant women being susceptible to prenatal psychological distress. Maternal prenatal distress is a broad term that encompasses a set of non-specific symptoms like anxiety, stress, and depression [1]. Various studies have shown that prenatal distress is responsible for adverse neonatal outcomes like pre-term birth, low birth weight, need for a cesarean section, intra-uterine growth restriction, neurodevelopmental delays, lower social, emotional, and cognitive abilities, infant anxiety and behavioral problems, delayed motor development [2], etc., thereby highlighting the importance of maternal psychological well-being from early pregnancy.

One of the factors affecting maternal psychological well-being is poor sleep, which predicts inadequate weight gain in mothers, fetal growth restriction, preterm birth, and low birth weight [3]. Literature suggests that vomiting episodes and BMI are closely related to psychological distress [4,5]. Studies show an association of prenatal distress with poor sleep quality [6]. We hypothesize that in this mutually dependent cycle of psychological distress and sleep disturbance, there are pregnancy-induced changes, thus making pregnancy a phase prone to distress. However, data on the assessment of the relationship and determinants of poor sleep quality and prenatal distress are scanty. Additionally, these factors are not sufficiently studied during early pregnancy during which their unaddressed impact could increase over the course of gestation and lead to adverse maternal and neonatal outcomes.

Geography and socioeconomic status are important predictors of prenatal distress. In a developing country, such as India, health disparities persist due to the wide socioeconomic strata divide among women from rural and urban areas [7]. It is seen that while rural women have relatively insufficient access to healthcare, lack of awareness relating to pregnancy, higher neonatal morbidity and mortality, etc.; urban women are more prone to develop pregnancy-related morbidities like gestational diabetes mellitus owing to higher body mass index (BMI) [8], eclampsia, need for cesarean delivery, overt medical skepticism, etc. This creates additional psychological distress among women which may compromise maternal nutrition, thereby hampering the growth of the child in utero and at birth. The prevalence of antenatal depression ranges in India from 6-48% [9], thus, identifying factors leading to psychological distress is of utmost clinical and social relevance.

Prenatal distress is a global public health concern. It is a part of the third sustainable development goal, Target 3.4, which aims to promote mental health and well-being by 2030, thus highlighting the urgent need to tackle psychological distress [10]. Due to the paucity of literature on early pregnancy distress and measures to regulate it, there is an increased risk of sub-optimal development of maternal and child health during pregnancy [11]. Studies conducted in this area have reported on linkages between post-natal depression and maternal-child health [12]. However, the post-natal period is a late stage to initiate interventions because a significant proportion of the infant’s growth and development has already occurred in utero.

Further, data on epidemiological evidence pertaining to sleep quality and maternal distress from low-middle-income countries, such as India, are scarce. Given that India is now one of the most populous nations globally, it is imperative to better characterize and address sleep quality and prenatal mental health, as it has long-term repercussions for maternal and child health. Thus, the objectives of our study were to assess the prevalence of poor sleep quality and prenatal distress during early pregnancy, and study determinants of poor sleep quality and prenatal distress during early pregnancy from urban and rural settings.

## Materials and methods

Study design and subjects

A baseline assessment was conducted in the form of a cross-sectional observational study on early pregnant women (gestational age of less than 12 weeks) from a cohort - MAI (Mother and Infant; MAI means “mother” in Marathi - a local language indigenous to the central state of Maharashtra). Data from participants of the MAI cohort were analyzed for the current study. The MAI cohort study is a longitudinal, prospective community-based cohort study conducted in the rural and urban areas in and around Pune City, Maharashtra, India. Longitudinal findings from the rural locus have been published earlier [13].

Sample size calculation

The sample size was calculated as 325 a priori, considering a 20% attrition rate, alpha 0.05, 0.8 power, and effect size f2 of 0.1 using a linear multiple regression fixed model (F test family) and G power software (Version 3.1.9.7; https://www.psychologie.hhu.de/arbeitsgruppen/allgemeine-psychologie-und-arbeitspsychologie/gpower). Post-hoc power calculation was performed for the objectives of the present paper with an alpha of 0.05, using the logistic regression test, and it was adequate to achieve a power of 0.8.

Rural and urban pregnant women attending primary, secondary, and tertiary health care centers and private outpatient department clinics were screened and approached for the study in person and followed up on the phone with their due permission.

Inclusion and exclusion criteria

**Figure 1:**
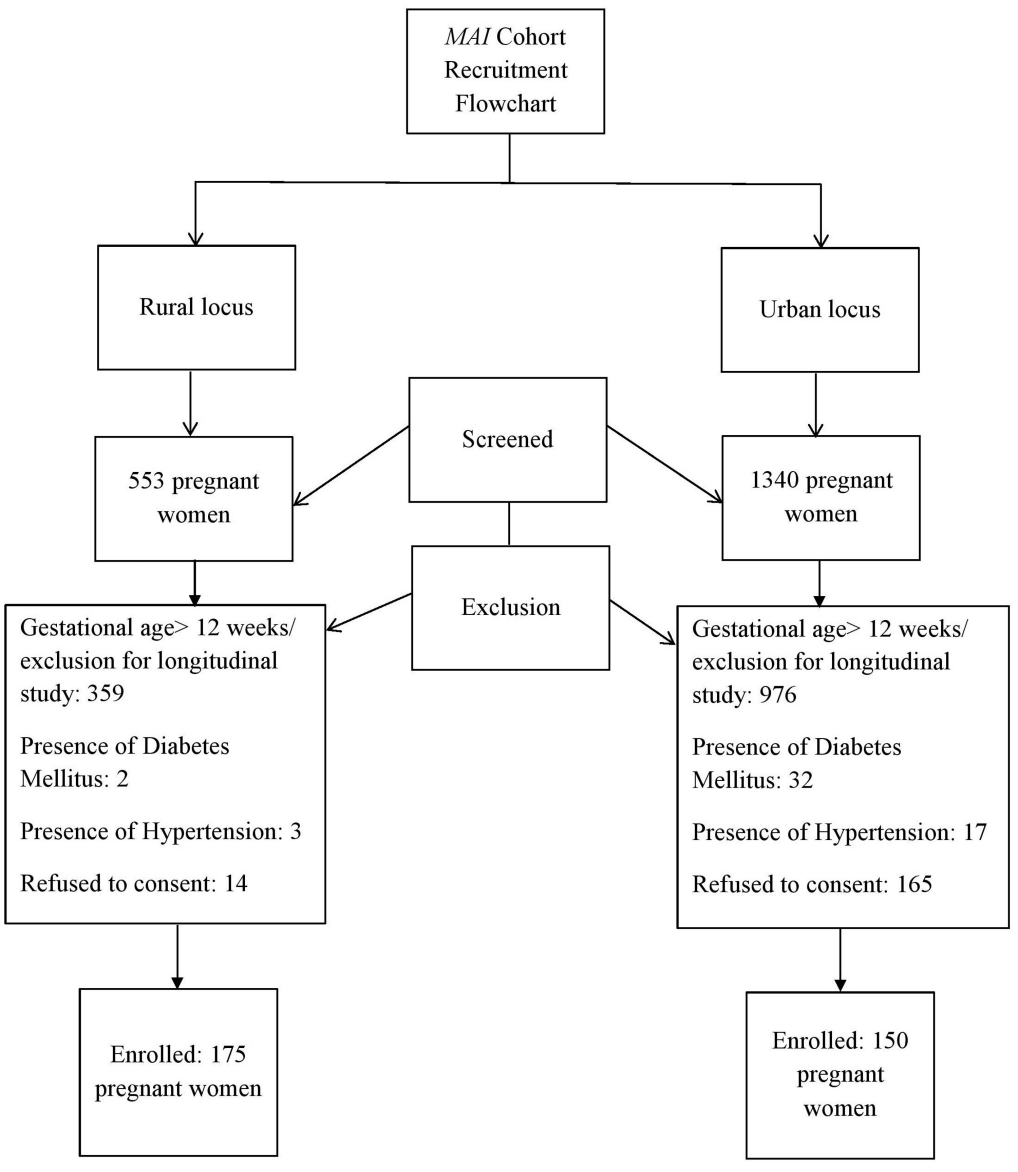
MAI (Mother and Infant) cohort recruitment flowchart

Apparently healthy pregnant women of reproductive age with an absence of pre-existing co-morbidities, such as diabetes mellitus and hypertension, with gestational age between 8-12 weeks, i.e., the first trimester, and those who agreed to participate in the study by consenting were enrolled in the study. The screening and enrolment process has been described in brief in Figure 1. The recruitment percentage for the rural locus was 92.5%, whereas that for the urban was 47.6%, thus accounting for the total recruitment rate to be 64.4%.

Data collection

Data collection was performed from August 2020 to March 2023. Ethics approval was obtained from the institutional ethics committee of Jehangir Clinical Development Center (Registration number: JCDC/BHR/23/048) (Dated: 02/03/2020). The study protocol was explained in detail and written informed consent was obtained before any study procedures were performed.

Maternal pre-pregnancy BMI was calculated based on height and weight recorded on the day of enrolment on standard calibrated instruments and was categorized as per the World Health Organization definition. Data on family socioeconomic status (SES) were recorded using the New Socioeconomic Classification system according to the Socioeconomic Classification 2011 [14]. Maternal demography, clinical history, and reproductive history were collected using the interview method. Nulliparous women were defined as those having no previous births.

Data on maternal sleep quality was assessed using the Pittsburgh Sleep Quality Index (PSQI), a reliable tool (Cronbach’s alpha: 0.83) for measuring subjective sleep. PSQI measures self-reported quality and patterns of sleep, where good sleep vs. poor sleep is differentiated based on seven domains: sleep latency, habitual sleep efficiency, subjective sleep quality, sleep duration, use of medication, sleep disturbance, and daytime dysfunction due to sleepiness over the past month. The domains range from 0 to 3, where 0 indicates good sleep and 3 indicates poor sleep. Based on these domains, an average sum - the global sleep quality score was calculated, where a value of equal to and more than 5 indicates poor sleep [15].

For assessing prenatal distress, the revised Prenatal Distress Questionnaire (NuPDQ), developed by Lobel M and team, was used. The questionnaire is based on the 17-item 3-point Likert scale (1 = no distress to 3 = high distress) across sub-scales (concerns about the baby, physical and social changes during pregnancy, concerns related to childbirth, healthcare quality and health status, concerns about finances, care of the baby and post-partum life). The global score ranged from 17 to 51, where higher scores indicate higher distress [16]. Tertiles were formulated since there are no established cut-offs, thus, scores ranging from 17 to 24 were coded as low distress, 25 to 32 as medium distress, and 33 and higher as high distress. Questionnaires were translated into the local regional language (Marathi), and the interview was administered to pregnant women by bilingual researchers. Due permissions to use questionnaires were obtained a priory from respective authors through personal correspondence.

Statistical analysis

Statistical analysis was performed using Statistical Package for Social Sciences (IBM SPSS) version 27 (. Prenatal distress score and sleep quality score were non-normally distributed; and have been expressed as median and inter-quartile range (IQR). Categorical variables were expressed in percentages. The Mann-Whitney test was used to test the significance in differences of sleep quality and prenatal distress across locations. Since there were no established cut-offs available, we categorized prenatal distress as: low (score: 17-24), medium (score: 25-32), and high distress (score: 33 and above) (global score ranged from 17-51) based on tertiles. We performed binary logistic regression to predict determinants of poor sleep quality. Independent variables were location of residence, BMI categories (underweight, normal, overweight/obese), and complaints of episodes of vomiting and prenatal distress. Multinomial logistic regression was used to predict determinants of prenatal distress, where the low prenatal distress category was the reference category. Independent variables were location of residence, BMI categories (underweight, normal, overweight/obese), complaints of episodes of vomiting, and sleep quality. We adjusted both the models for maternal age, reproductive history - gravidity (number of times the woman has been pregnant) and socioeconomic status. A p-value of <0.05 was considered statistically significant.

## Results

The study population included pregnant women from rural (n=175) and urban (n=150) settings. Rural women were significantly shorter and lighter as compared to their urban counterparts (p<0.05) (Table 1).

**Table 1:** Sociodemographic characteristics of rural and urban pregnant women IQR: inter-quartile range, BMI: body mass index *Values significantly different from urban counterparts The chi-square test was used to test the significance of differences across locations for categorical variables and the Mann-Whitney test for continuous variables.

Parameter	Rural (n=175)	Urban (n=150)	Total (n=325)	p-value
Age at pregnancy (years) Median (IQR)	22.7 (20.5-25.6)*	30.6 (28.8-33.3)	27.1 (22.2-30.8)	0.001
Age at marriage (years) Median (IQR)	19.9 (18.4-22.1)*	26.3 (24.2-28.1)	23.0 (19.5-26.5)	0.001
Gestational age at enrolment (weeks) Median (IQR)	10 (8-11)	11 (9-12)	9 (10-12)	0.100
Socioeconomic status (N (%))				
Low socioeconomic status	57 (32.6)*	-	57 (17.5)	0.001
High socioeconomic status	118 (67.4)*	150 (100)	268 (82.5)	0.030
Education (N (%))				
10^th^ grade/ 12^th^ grade	124 (70.9)*	10 (6.7)	134 (41.2)	0.001
Graduate/Postgraduate	51 (29.1)*	140 (93.3)	191 (58.8)	0.001
Maternal height (cm) Median (IQR)	153.7 (149.7-157.5)*	155.7 (152.5-159.2)	154.8 (150.7-1585)	0.001
Maternal weight (kg) Median (IQR)	46.0 (41.2-53.6)*	61.4 (54.8-68.2)	53.4 (44.5-63.6)	0.001
Pre-pregnancy BMI (kg/m^2^) Median (IQR)	19.5 (17.8-22.0)*	25.3 (22.8-28.5)	22.2 (18.6-26.3)	0.001
Pre-pregnancy BMI categories (N (%))				
Underweight	67 (38.3)*	11 (7.3)	78 (24)	0.001
Normal	84 (48)*	54 (36)	138 (42.5)	0.001
Overweight	17 (9.7)*	65 (43.3)	82 (25.2)	0.001
Obese	7 (4)*	20 (13.3)	27 (8.3)	0.001

Reproductive history and physical symptoms of pregnancy

In terms of reproductive history, 51.1% of women were nulliparous, in 48.9%, gravidity was more than one, and 23.1% had borne one or more live infants in the past. Forty-nine point eight percent (49.8%) of women (59.4% rural women as compared to 38.7% urban women) complained of episodes of vomiting during early pregnancy (p<0.05). Sixty-three point four percent (63.4%) of women complained of nausea (69.1% vs. 56.7%) and 42.5% (44.6% vs. 40%) women complained of heart-burn during early pregnancy of which a majority were significantly prevalent in rural women as compared to their urban counterparts (p<0.05).

Prevalence of poor sleep quality and prenatal distress

Rural women had significantly poor sleep component scores of sleep latency, sleep disturbance, and sleep duration (p<0.05) (Table 2). Forty-five point five percent (45.5%) of women experienced bad sleep quality. Rural women (51.4%) experienced significantly poor sleep quality as compared to urban women (38.7%) (p < 0.05). The prevalence of prenatal distress in the study was 37.5%, irrespective of location. Prevalence was significantly higher in rural women (40%) (mean score: 31.4±7.5) as compared to urban women (34.7%) (mean score: 29.1±10.6) (p<0.05). Low distress was seen in 45.3% of urban women and 18.3% of rural women (p<0.05).

**Table 2:** Sleep quality and prenatal distress of urban and rural pregnant women *Values significantly different from urban counterparts $Values range from 5 to 21, where <=5= Best, >5= Poor #Value ranges from 0 to 3, where 0=Best, 3= Poor Mann Whitney test was used to test the significance in differences of sleep quality and prenatal distress across locations

Parameter	Rural (n=175)	Urban (n=150)	Total (n=325)	p-value
Prenatal distress score Median (IQR)	31 (26-37)*	25 (19.7-41)	30 (23-37.5)	0.001
^$^Pittsburgh sleep quality index score (PSQI) Median (IQR)	5 (3-8)*	4 (2-6)	4 (3-7)	0.002
^#^PSQI component scores Median (IQR):				
Sleep duration	0 (0-1)	1 (0-0.2)	0 (0-1)	0.10
Sleep disturbance	1 (1-2)*	1 (0-1)	1 (1-1)	0.001
Sleep latency	2 (0-2)*	1 (0-2)	1 (0-2)	0.001
Sleep efficiency	0 (0-1)	0 (0-1)	0 (0-1)	1.00
Day dysfunction due to sleepiness	0 (0-2)*	2 (0-3)	1 (0-2)	0.001
Need medication for sleep	0(0-0)	0 (0-0)	0 (0-0)	0.12
Overall sleep quality	0 (0-1)*	0 (0-0)	0 (0-1)	0.001
Pittsburgh sleep quality index (n (%))				
Poor sleep	77 (44)*	72 (48)	149 (45.8)	0.01
Good sleep	98 (56)*	78 (52)	176 (54.2)	0.01

Relationship between prenatal distress and poor sleep quality

**Figure 2:**
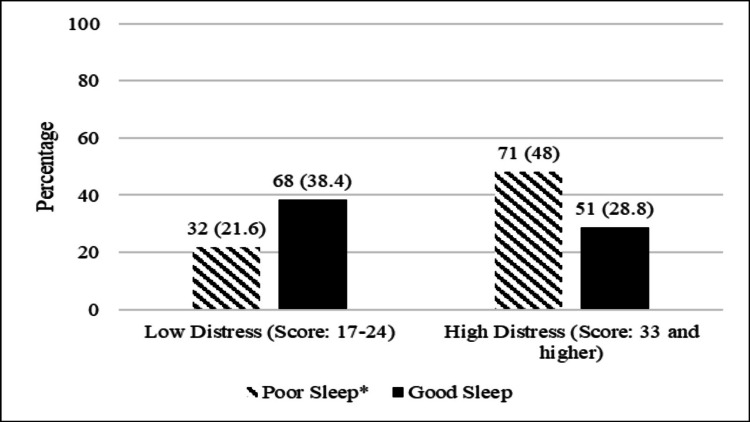
Sleep quality according to prenatal distress (low distress: score ranging from 17-24, high distress: score ranging from 33 and higher); (poor sleep: score of ≥5, good sleep: score of <5)

We found that irrespective of location, pregnant women with low distress had significantly good sleep quality. About half the pregnant women who experienced high distress also experienced significantly poor sleep (p<0.05) (Figure 2). To further explore the relationship between sleep quality and prenatal distress, we computed correlations between the two and found that high distress showed a significant moderate correlation to poor sleep quality (ρ = 0.308, p = 0.001).

Predictors of poor sleep quality

After adjusting the model for maternal age, gravidity, and socioeconomic status, binary logistic regression revealed that the only significant contributor to poor sleep quality among rural and urban pregnant women during early pregnancy was high prenatal distress (B=2.63, 95% CI: 1.47-4.69, p = 0.001) and not medium prenatal distress (B=1.34, 95% CI: 0.72-2.48. p = 0.35). 

Predictors of prenatal distress

Multinomial logistic regression was used to predict determinants of prenatal distress among rural and urban pregnant women. The results from Table 3 suggest that rural women were more likely to suffer from prenatal distress (OR: 6.37, 95% CI: 2.46-16.51). Mothers with BMI at extreme ends of the spectrum, i.e., underweight (OR: 2.21, 95% CI: 0.97-5.05) and those who were overweight/obese (OR: 1.19, 95% CI: 0.57-2.50) were observed to be more prone to prenatal distress; however, the difference was not statistically significant. Women who experienced episodes of vomiting experienced high distress (OR: 1.91, 95% CI: 1.07-3.40) (p<0.05). Pregnant women who experienced good sleep were significantly less likely to experience high prenatal distress (OR: 0.37, 95% CI: 0.21-0.67) (p<0.05).

**Table 3:** Determinants of prenatal distress among pregnant women a The reference category is low prenatal distress. B: beta coefficient, SE: standard error, OR: odd’s ratio, CI: confidence interval Multinomial logistic regression was carried out to generate the predictors of medium and high prenatal distress.

Variable	B	SE	Wald	p-value	OR (95% CI)
^a^ Medium prenatal distress					
Maternal age	0.05	0.04	1.26	0.26	1.05 (0.96-1.14)
Gravidity	-0.02	0.17	0.02	0.88	0.97 (0.69-1.36)
Socioeconomic status:					
Low	-0.31	0.46	0.46	0.49	0.73 (0.29-1.81)
High	Reference				
Location of residence					
Rural	1.85	0.48	14.57	0.0001	6.37 (2.46-16.51)
Urban	Reference				
Pre-pregnancy BMI					
Underweight BMI	0.79	0.42	3.56	0.05	2.21 (0.97-5.05)
Overweight/ Obese BMI	0.17	0.37	0.22	0.61	1.19 (0.57-2.50)
Normal BMI	Reference				
Reported episodes of vomiting					
Yes	0.53	0.30	3.00	0.08	1.70 (0.93-3.13)
No	Reference				
Sleep quality					
Good	-0.29	0.31	0.87	0.35	0.74 (0.40-1.38)
Poor	Reference				
^a^ High prenatal distress					
Maternal age	0.03	0.04	0.72	0.39	1.03 (0.95-1.12)
Gravidity	-0.06	0.16	0.17	0.68	0.93 (0.67-1.28)
Socioeconomic status					
Low	0.03	0.46	0.004	0.94	1.03 (0.41-2.55)
High	Reference				
Location of residence					
Rural	0.85	0.45	3.47	0.06	2.34 (0.95-5.76)
Urban	Reference				
Pre-pregnancy BMI					
Underweight BMI	0.30	0.41	0.52	0.46	1.35 (0.59-3.07)
Overweight/Obese BMI	-0.39	0.34	1.28	0.25	0.67 (0.34-1.33)
Normal BMI	Reference				
Reported episodes of vomiting					
Yes	0.64	0.29	4.86	0.02	1.91 (1.07-3.40)
No	Reference				
Sleep quality					
Better	-0.97	0.29	10.74	0.001	0.37 (0.21-0.67)
Poor	Reference				

## Discussion

**Figure 3:**
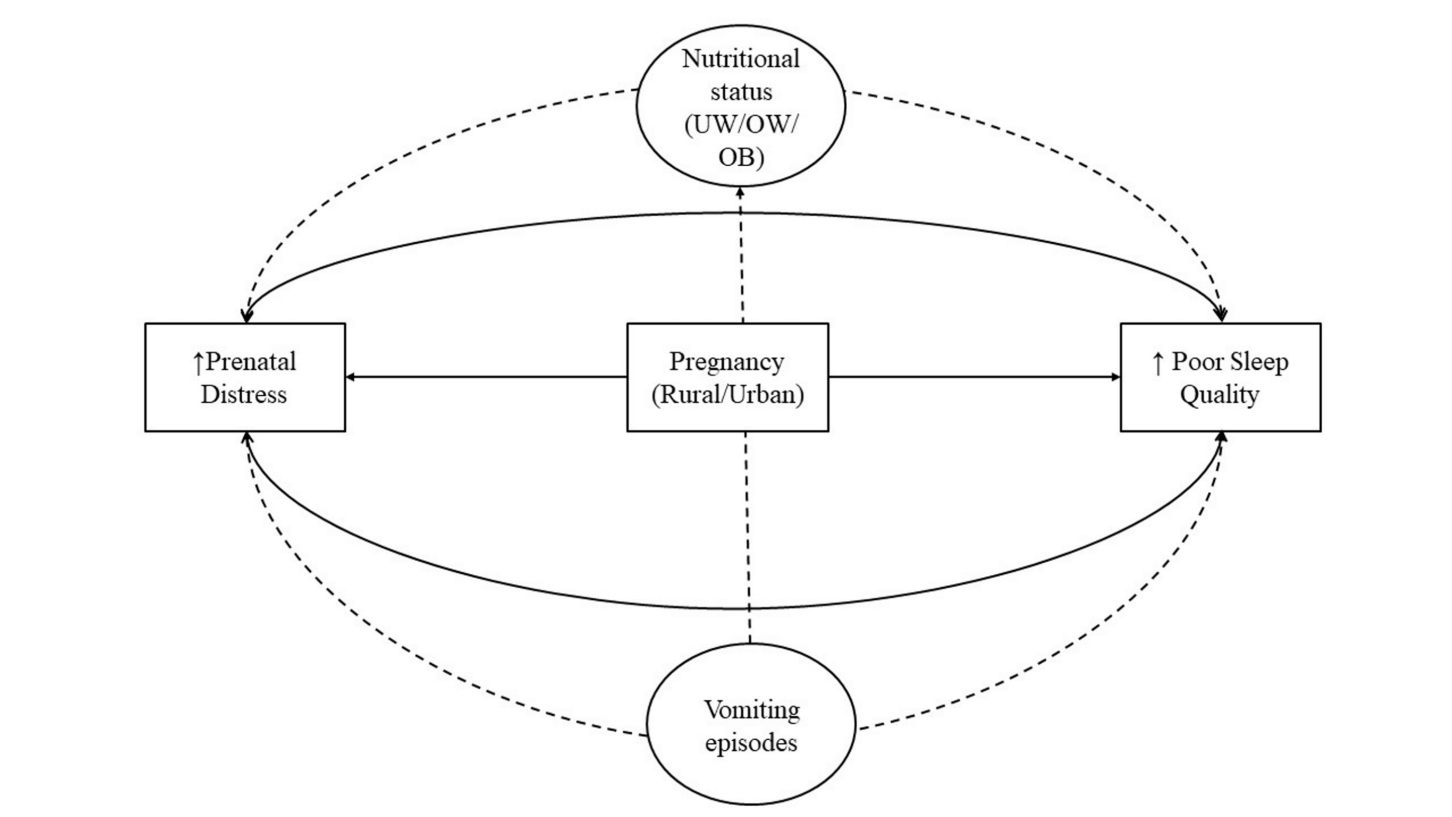
Mutual dependencies between poor sleep quality and prenatal distress during early pregnancy UW: underweight BMI, OW: overweight BMI, OB: obese BMI Image credits: MD, NS, NK, AK

Our study, which analyzed data on 325 pregnant women during early pregnancy (i.e., first trimester) found that sleep quality was altered during early pregnancy; 45.5% of women experienced bad sleep quality, whereas rural women were significantly more prone to experiencing sleep disturbances, longer latency, and shorter duration. A high prevalence of prenatal distress among pregnant women (almost one-third of the sample) was reported in our sample. Consequently, it was observed that women residing in rural areas were six times more likely to experience prenatal distress as compared to their urban counterparts. Poor sleep quality was a significant contributor/predictor of prenatal distress and vice versa, thus highlighting a mutually dependent relationship between the two. Finally, a key finding of our study was the impact of episodes of vomiting reported during the first trimester on prenatal distress, which was again predominantly observed among rural women; however, urban women experienced it too (Figure 3).

Disturbed sleep and overall poor sleep quality often complicate psychological distress. In a study by Zaky et al., the prevalence of bad sleep quality was similar to ours (40%) [3]. In a study conducted by Venugopal et al., the prevalence of bad sleep quality was 24.4% [17]; however, the prevalence reported in our study is twice what they found. This could be attributed to a smaller sample size in their study and an average plotted prevalence over all three trimesters of pregnancies, without accounting for trimester-specific prevalence. We found that prenatal distress was a significant contributor to poor sleep quality. It is found that sleep quality decreases with an increase in gestational age; therefore, early screening for sleep disorders and identifying its predictors are of utmost importance.

Prenatal distress, specifically prenatal stress, is known to be associated with higher circulating levels of tumor necrosis factor and pro-inflammatory cytokines like interleukin 1b and 6, which are studied to have been related to the development of central nervous system dysfunction and thus, the probability of developing behavioral and mental health disorders in later life [18]. Progression of prenatal distress into prenatal depression is often quick if there is no intervention [1]. Ignorance to paying attention to pregnant women’s mental health is observed particularly in rural areas where being mentally unwell is considered a stigma [19].

We found that women residing in rural areas had a higher prevalence of distress. Similarly, Fisher et al., in their meta-analyses, also observed that women residing in rural areas from a low- and middle-income country (LMIC) setting are more prone to prenatal distress as compared to those who live in an urban non-LMIC setting [20]. In a study by Tang et al., the reported prevalence of prenatal stress, anxiety, and distress was 9.2%, 15%, and 5.1%, respectively in areas of low socioeconomic status [11]. These findings are supported by existing literature [21]. All the above studies corroborate the findings presented in the current study, i.e., women from rural areas are more prone to develop prenatal distress as compared to their urban counterparts. This may be because rural women experience relatively less access to medical care, there is a lack of awareness and more emphasis on physical health rather than emotional well-being [22]. Addressing this problem is of utmost priority since poor mental well-being is one of the determinants of fetal growth restriction in utero and at birth [23].

In our study, we found that women at extreme ends of the BMI spectrum were more prone to high prenatal distress; however, the effect was not statistically significant. This is in contrast to what Arora and Aeri [24] found that obese women were at the highest risk of experiencing depression throughout pregnancy as compared to women with normal or overweight BMI. Another key finding noted in our study was that the symptoms of episodes of vomiting were a strong, significant predictor of prenatal distress. Similar findings were reported in the study by Kjeldgaard et al. [4].

In a study conducted by Gao et al., it was found that poor sleep quality was positively associated with antenatal distress (OR: 2.60, CI: 1.79-3.77), which is similar to the findings of our study [25]. In another study, the authors found that 87% of women experienced bad sleep quality [26], whereas we observed the numbers to be 45.5%. This could be attributed to the fact that a mere 4.8% of their study population were in the first trimester and the remaining were from the second and third trimesters. It has been hypothesized that distress increases with an increase in gestational age. Their study also stated that prenatal distress was one of the determinants of poor sleep quality. Similar to our study, they also found a cyclical relationship between sleep and distress in that bad sleep quality was an important indicator for prenatal distress, which itself may lead to bad sleep quality. The reported underlying biological mechanisms for this are uncertain. A study reports that bad sleep is a significant contributor to chronic inflammation, which causes dysregulation of melatonin, in turn, increasing perceived stress [27]. They also report that women who have been subjected to disturbed sleep or increased sleep latency experience gradual alteration in neurotransmitter receptor systems, including norepinephrine and 5-hydroxytryptophan, which are responsible for the functioning of mental, emotional, and cognitive well-being; similar to the pattern observed in individuals with major depression.

As mentioned earlier, distress can quickly progress into depression if appropriate measures are not taken. It was observed that in the year 2013, the point prevalence of prenatal depression in India was 15.5% [28]. This has increased to 25.5% in 2023 according to a systematic review by Mitchell et al. (2023) [29]. This 65% rise in prenatal depression over the last decade puts India in a challenging position to achieve SDG 3, indicator 3.4 (promote mental health and well-being) by the year 2030. In our cohort, we observed an even higher percentage of pregnant women experiencing prenatal distress (37.5%), with rural women suffering more as compared to their urban counterparts. A study has reported a similar prevalence of prenatal distress; however, it enrolled women from later stages of pregnancy, which becomes a challenging phase to diagnose and start interventions, thus highlighting the need to screen for prenatal distress earlier in pregnancy [30].

Nonetheless, despite mental health awareness campaigns being conducted all over, we see that mental health is still one of the low-priority areas in the field of health and well-being. Future recommendations need to focus on campaigns of mental health screening and referrals to specialized psychologists, along with mental health awareness campaigns, thus completing the otherwise incomplete loop. This could help India move one step closer to achieving SDG 3.

Due to the paucity of data, ours is one of the handful of studies that takes into account differential prevalences of poor sleep quality and prenatal distress during early pregnancy in urban and rural settings and factors associated thereof, which was performed in an LMIC setting such as India. A larger sample size would likely have improved the strength of the association of factors affecting high vs. low prenatal distress; however, our sample size was adequate for the results to be generalized and is comparable with other studies. Our strength is also the use of validated and reliable tools for measuring early prenatal distress and sleep quality. We have taken into account anthropometry, socioeconomic status, and clinical history - reproductive history and sleep while measuring prenatal distress. We feel this is a well-rounded approach to translating research findings into clinical practice and social relevance. Since we performed assessments in the first trimester, similar assessments performed clinically may give a clinician time to work on prenatal distress and possibly improve pregnancy outcomes.

Our study was limited by the cross-sectional design. Owing to the interdependence between prenatal distress and poor sleep quality, a cross-sectional design is often inadequate to explain mutual dependencies; therefore, longitudinal studies need to be planned to establish a cause-effect relationship. Following up on the women till they deliver may help throw more light on the impact of distress on pregnancy outcomes. Another area of improvement could have been the use of objective measures of assessing sleep quality and prenatal distress using polysomnograms and electroencephalography, respectively, for the sake of precision. However, these measures would have been invasive and thus, we used non-invasive subjective perceived measures to estimate sleep quality and distress, which, from the available literature, are fairly accurate.

## Conclusions

Our present study has provided evidence regarding the high prevalence of poor sleep quality and prenatal distress across Indian rural and urban women in the first trimester. We reported that high prenatal distress contributed to poor sleep quality. We found that rural underweight pregnant women who experienced symptoms of episodes of vomiting and bad sleep quality were at a higher risk of developing prenatal distress as compared to urban women. We also found a relationship between sleep and distress, paving the way for further researchers to explore the clinical and social aspects of this phenomenon, for managing and preventing the progression of poor sleep quality and prenatal distress. This study underlines the need for interventions to manage poor sleep quality and prenatal distress during early pregnancy in order to avoid adverse maternal and neonatal outcomes.
